# Peptide-Mediated Immobilization on Magnetoferritin for Enzyme Recycling

**DOI:** 10.3390/nano9111558

**Published:** 2019-11-02

**Authors:** Yu Zhang, Yixin Dong, Jinhua Zhou, Ying’ao Hu, Xun Li, Fei Wang

**Affiliations:** 1 Jiangsu Provincial Key Lab for the Chemistry and Utilization of Ago-Forest Biomass, College of Chemical Engineering, Nanjing Forestry University, Nanjing 210037, China; kaydong417@163.com (Y.D.); 13770559414@163.com (J.Z.); hya8147213@163.com (Y.H.); xunlee@njfu.edu.cn (X.L.); hgwf@njfu.edu.cn (F.W.); 2 Jiangsu Co-Innovation Center of Efficient Processing and Utilization of Forest Resources, Nanjing Forestry University, Nanjing 210037, China

**Keywords:** magnetoferritin, enzyme immobilization, coiled coils, nanoparticles, recycling

## Abstract

Ferritin possess favorable properties because its exterior and interior surface can be applied to generate functional nanomaterials, which make them possible for enzyme immobilization and recycling. Here, we report the noncovalent immobilization of a genetically modified *β*-glucosidase onto the outer surface of synthetic magnetoferritin through the electrostatic interaction of a heterodimeric coiled-coil protein formed by coils containing lysine residues (K-coils) and coils containing glutamic acid (E-coils). The immobilized enzyme was characterized, and its enzymatic properties were evaluated. Furthermore, reusability of immobilized enzyme was demonstrated in aqueous solution under an applied magnetic field. The results showed that magnetoferritin was successfully prepared and it was an excellent support for enzyme immobilization. After three times usages, the retention rates were 93.75%, 82.5%, and 56.25%, respectively, demonstrating that immobilized enzyme possessed good retention efficiency and could be used as potential carrier for other biomolecules. The strategy of enzyme immobilization developed in this work can be applied, in general, to many other target molecules.

## 1. Introduction

In biotechnological processes, the preparation of immobilized enzymes is a key step in optimizing the operational performance of an enzyme in aqueous or non-aqueous media. The most frequently used enzyme immobilization techniques are noncovalent adsorption or deposition [1,2], covalent attachment [3,4], entrapment in a polymeric device [5,6], and enzyme cross-linking [7,8]. To some extent, the recovery and recycling of an immobilized enzyme can lower expenses, however, immobilization processes can suffer from low enzyme loading, weak binding ability and difficulties in keeping the enzyme fixed to the carrier, and limited enzyme recovery. New techniques are needed to avoid such issues. In one particularly promising strategy, ferritin nanoparticles based on magnetite (Fe_3_O_4_) and maghemite (γ-Fe_2_O_3_), referred to as magnetoferritin, are used as the supporting material for enzyme immobilization. This material protects the enzyme and allows for simple recovery through the application of a magnetic field.

Ferritin is an iron storage protein with a spherical structure that plays an important role in regulating cellular iron content. Channels created by the symmetrical self-assembly of ferritin monomers into cage-like structures can guide Fe(II) ions toward catalytic centers [9]. Twenty-four identical polypeptide subunits assemble into a ferritin cage with an outer diameter of 12 nm and an inner diameter of 8 nm. This protein shell is capable of storing up to 4500 Fe(III) atoms [10,11]. Furthermore, apoferritin, which is void of iron ions in the cavity, can be used to synthesize Fe oxide magnetic nanoparticles, referred to as magnetoferritin [12]. Magnetoferritin was chosen as a possible paramagnetic nanoparticle due to its homogeneous dispersion in aqueous solution, sub-10 nm feature size, and response to magnetic filtration. Magnetoferritin is also relatively biocompatible and flexible, and has been used in a variety of biomedical applications [13], magnetic resonance imaging [14], and for radioactive ion separation in water treatment [15]. In addition, magnetoferritin nanoparticles can be arranged into well-ordered periodic arrays free of unwanted aggregation [16]. The immobilization of enzymes onto nanosized magnetic ferritin particles would allow for better separation from a reaction mixture with the use of an external magnetic field, thereby, simplifying reuse and recycling.

**Figure 1:**
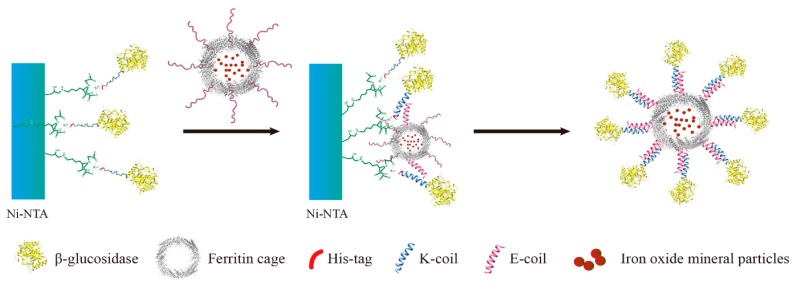
Purification of *β*-glucosidase and the human H chain ferritin without the sequence of a His-tag (KG-HE) protein complex. A solution containing the N-terminus of *β*-glucosidase (KG) is added to a Ni-nitrilotriacetic acid (Ni-NTA) column. Only KG binds to the Ni-NTA with its N-terminal His-tag (sequence of KG was ‘(N-terminal) His-tag–K-coil–glucosidase (C-terminal)’). A washing step removes all proteins lacking the His-tag. The solution containing the magnetoferritin of human H chain ferritin without the sequence of a His-tag (HE) is then added. The HE with the C-terminal coiled coil (pink) binds to the N-terminal coiled coil (blue) of KG. After another wash step, the KG-HE complex is eluted from the column using an excess of imidazole.

A novel immobilization method, employing a noncovalent anchoring moiety to link the enzyme to the exterior surface of magnetic ferritin, was demonstrated herein and is shown in Figure 1. The coiled coil is a protein structural motif consisting of two or more α-helices that are wrapped around each other in a superhelical fashion. The primary structure of coiled coil-forming proteins is characterized by a distinct seven-residue repeat, denoted as (abcdefg)_n_ where n is the number of repeats. Positions “a” and “d” are occupied by hydrophobic residues that form the hydrophobic core of coiled coils, whereas positions “e” and “g” contain complementary charged side-chain functional groups that interact to form stabilizing salt bridges. The motivation of burying hydrophobic faces provides the primary driving force for self-assembly. Charged residues beside the hydrophobic faces can provide additional stability by forming intra- or interhelical salt bridges, and can help match the orientation of interacting peptide chains [17]. Electrostatic interactions can play an important role in the heterodimer of a coiled-coil structure [18,19], however, for our purposes, we employed a heterodimeric coiled coil to avoid the dimerization of ferritins or enzymes. To eliminate the need to chemically link two proteins, a heterodimeric coiled-coil protein, consisting of E- and K-coils and having a small dissociation constant (K_d_ = 7 × 10^−8^ M), was used as a noncovalent anchor [20]. In this study, heterodimer formation was achieved by the placement of charged residues at the “e” and “g” positions of the heptad repeat; the E-coil contains glutamic acid residues, and the K-coil contains lysine residues, at these respective positions. Since the outer surface of ferritin exhibits a relatively low isoelectric point (pI = 5.3) [21], the negatively charged E-coil was chosen as the attachment point for ferritin, ensuring that the coiled coil does not stick to the ferritin surface, and thus making it more efficient with respect to binding the K-coil. In this study, the complete amino acid sequences of the two peptides were (EIAALEK)_3_ for the E-coil linked to human H chain ferritin and (KIAALKE)_3_ for the K-coil linked to *β*-glucosidase (Table 1).

The carboxyl terminus, including the E-helix, is not essential for proper folding of the monomer subunit and assembly of ferritin [22]. Interestingly, human H chain ferritin nanocages can assemble into two conformations: “flip”, where the E helix of the carboxy terminus points toward the cavity, and “flop”, where the E-helix of the carboxyl terminus points outside [22]. In general, the flip conformation is more common than the flop conformation in the construction of a native ferritin shell, however, fusion of the C-terminus of human H chain ferritin with a certain peptide or protein will result in a flop conformation, according to the observations of Cesareni et al. [22]. Whether the conformation is flip or flop depends on whether the volume inside the cage is sufficient to contain the 24 C-terminal peptides [23]. Thus, it is possible to attach enzymes to the outer surface of the ferritin by exploiting the electrostatic interactions between the E- and K-coils.

The objective of this work was to immobilize enzyme onto the outer surface of the magnetoferritin through the heterodimeric coiled-coils and recycle the enzyme under the magnetic field. Because the E-coil binds to the K-coil in a parallel fashion, plasmids of this E and K fusion systems were designed separately, and an E-coil was introduced into the C-terminus of human H chain ferritin without the sequence of a His-tag (named HE). In addition, a K-coil was introduced to the N-terminus of *β*-glucosidase (named KG), which contains an N-terminal His-tag (Table S1). Magnetoferritin of HE was synthesized and characterized and KG was immobilized onto the surface of the magnetoferritin of HE. The enzymatic properties and kinetics of KG and KG-HE were compared which showed that the immobilization has almost no effect on enzymes. Under the magnetic field, immobilized enzyme showed good retention efficiency after three times usages. The developed immobilization strategy can be applied to many other objectives.

## 2. Materials and Methods

### 2.1. Construction of HE and KG in Vector pET-20b

The original sequence of human H chain ferritin (HFtn) was synthesized previously in our lab. In order to construct the genetically engineered HFtn with the C-terminal E-coil sequence was designed, and the E-coil was fused to the C-terminus of HFtn using polymerase chain reaction (PCR).

The pET-20b vector containing the human H chain ferritin sequence was used as the template. To obtain plasmid of HE, primers were designed according to the sequence of target protein. These designed primers (Table S1) were synthesized and used for PCR. After PCR amplification, the products were purified by agarose gel electrophoresis. The chains containing the target gene were obtained and the cyclization was performed using T4 DNA ligase (TaKaRa, Dalian, China). The resulting plasmids were transformed into *E. coli* Top10 cells, and the DNA was extracted and the sequence of the HFtn with C-terminal E-coil was confirmed by DNA sequencing. The plasmid was then transformed into *E. coli* BL21 (DE3) cells, which were used for the production of the HFtn with E-coil (HE).

The primers for the construction of the *β*-glucosidase with N-terminal K-coil sequence were designed based on the sequences of *β*-glucosidase and K-coil (Table S1). The KG gene was amplified using PCR technique. Agarose gel electrophoresis was used to purify the PCR product. The plasmids were transformed into *E. coli* Top10 cells, and the sequence of the *β*-glucosidase with N-terminal K-coil (*KG*) was confirmed by DNA sequencing. The plasmids were transformed into *E. coli* BL21 (DE3) cells, which were used for the production of protein KG.

### 2.2. Expression of HE and KG

Plasmids harboring the gene of HE or KG were transformed into *E. coli* BL21 (DE3) (Novagen) cells. The LB medium (5 mL) containing 0.1 g/L of ampicillin was inoculated and incubated overnight at 150 rpm, 37 °C. The culture was then used to inoculate 200 mL of LB medium containing ampicillin (0.1 g/L) and grown at 37 °C. Protein expression was induced when OD_600_ reached 0.6 to 0.8, followed by addition of IPTG to a final concentration of 0.5 mM and incubated at 30 °C for 6 h.

*E. coli* cells containing HE or KG were harvested after expression by centrifugation at 5500× *g*, 4 °C for 5 min. The supernatant was discarded, and the pellet cells were resuspended in 8 mL lysis buffer (50 mM NaH_2_PO_4_, 10 mM imidazole and 300 mM NaCl, pH 8.0). The solution was sonicated for 5 s with a 5 s interval, and the process continued for 15 min. The samples were centrifuged at 8500× *g* for 30 min.

### 2.3. Purification of HE by Solid Ammonium Sulfate Precipitation

Solid ammonium sulfate (Nanjing Chemical Reagent Co., Ltd, Nanjing, China) was added into the supernatant of HE protein to make a final saturation of 20%. After stirring for 12 h at room temperature, precipitation was achieved and was harvested by centrifugation and the corresponding supernatant was added with solid ammonium sulfate again. The ammonium sulfate concentration was increased stepwise by 10% until the final saturation reached 90%.

### 2.4. Size Exclusion Chromatography (SEC) Analysis

To purify HE protein, the pellets harvested in ammonium sulfate precipitation were re-dissolved in PBS buffer (2 mM KH_2_PO_4_, 8 mM Na_2_HPO_4_, 136 mM NaCl, 2.6 mM KCl, and pH 7.4), and the solution which contained target protein was dialyzed against GFC buffer three times at 6 h intervals. Subsequently, the solution was centrifuged at 5500× *g* for 5 min to remove any denatured proteins. The supernatant was concentrated using the Amicon Ultra-4 10K centrifugal filter device (Millipore, Billerica, MA, USA). After centrifugation at 3000× *g* for 20 min, the target protein was purified by SEC. The samples were stored at 4 °C for later use.

KG was purified by Ni-NTA agarose (QIAGEN, Hilden, Germany) prior to the SEC analysis. Briefly, the supernatant, mentioned previously, was incubated with Ni-NTA agarose beads for 2 h at 4 °C to ensure that KG was bound to the beads. Subsequently, the flow-through was collected and the Ni-NTA column was washed with different concentrations of imidazole buffers. The purified KG was then eluted from the agarose beads by using elution buffer (50 mM NaH_2_PO_4_, 250 mM imidazole and 300 mM NaCl, and pH 8.0). The KG solution was dialyzed against GFC buffer for 24 h to remove the excess imidazole and a Amicon Ultra-4 10K centrifugal filter device was used to condense KG for further SEC analysis.

Proteins were further purified by SEC experiments on an ÄKTApurifier (GE Healthcare, Uppsala, Sweden) which was equipped with a Superdex^TM^ 200 10/300 GL column. The proteins were eluted by GFC buffer (50 mM Na_2_HPO_4_, 150 mM NaCl, and pH 7.2) with a flow rate of 0.5 mL min^−1^ as detected at 280 nm and 410 nm. The column was calibrated using six well-characterized protein standards, as previous described [9].

### 2.5. Synthesis and Characterization of Magnetoferritin

Magnetoferritin was synthesized using the method previously described [24]. Prior to transferring into the anaerobic chamber, all solutions were carefully deoxygenated with nitrogen. Solution of ferritin (1 mg/mL, 10 mL) dissolved in 100 mM Tris-HCl was added to the reaction vessel. The temperature of the vessel was maintained at 65 °C, and the pH value was stabilized at 8.5 that was adjusted by 100 mM NaOH. Freshly prepared H_2_O_2_ (4.17 mM) and Fe(II) (12.5 mM (NH_4_)_2_Fe(SO_4_)_2_∙6H_2_O) were added into the vessel with the stoichiometric equivalents (1:3 = H_2_O_2_: Fe^2+^) according to the equation below:

3Fe^2+^ + H_2_O_2_ + 2H_2_O → Fe_3_O_4_ + 6H^+^

After 50 min, 300 mM sodium citrate was added to chelate additional Fe^2+^. To remove the extra Fe irons precipitated outside the protein shell and the HE clusters, the synthesized ferrimagnetic HE was further purified to acquire the intact protein cages. Furthermore, the sample was centrifuged for 10 min at 8500× *g* to remove the aggregates, followed by gel filtration using Superdex^TM^ 200 10/300 GL as a column.

#### 2.5.1. Sucrose Density Gradient (SDG)

Sucrose density gradient (SDG) ultracentrifugation was performed to purify the synthesized magnetoferritin. A total gradient volume of solution (4 mL) containing magnetoferritin (1.23 mL) and sucrose gradients (20%, 35%, 50%, and 65%) were added to the centrifugation tube accordingly. Centrifugation was carried out at 2000× *g* for 5 h, at 4 °C. Distribution of magnetoferritin was determined by analysis of the absorption at 280 nm and 410 nm.

#### 2.5.2. TEM Analysis

Transmission electron microscopy (TEM) was used for further analysis. A small volume of Fe_3_O_4_-ferritin solution was placed onto a carbon-coated copper grid for TEM observation using JEM-1200EX (JEOL, Tokyo, Japan) with and without 1% uranyl acetate.

#### 2.5.3. HRTEM and SAED Analysis

A Tecnai G2 F20 S-TWIN (200 KV) was used for high resolution transmission electronic microscopy (HRTEM) analysis. The magnetoferritin samples were dried on carbon-coated copper grid for TEM observations. To prevent electron beam damage, all observations and image acquisitions were performed using exposures of <1 s. Selected area electron diffraction (SAED) patterns and lattice imaging were recorded on Tecnai G2 F20 S-TWIN (200 kV) (FEI, Hillsboro, OR, USA).

#### 2.5.4. Raman Spectra

A dried sample was prepared by freezing in liquid nitrogen and subsequently lyophilized for 12 h to 24 h. The 532 nm cw laser line irradiated through the sample of magnetoferritin to detect the intensities of the radiation which corresponded to the different wavelengths, and the Raman spectrum was collected by laser Raman spectrometer DXR532 (Thermo Fisher Scientific Inc., Asheville, NC, USA).

### 2.6. Preparation of KG-HE Complex

The E-coil is attached to the C-terminus of HE, which was added in excess to the column containing bound KG. This allows KE to bind to the modified *β*-glucosidase. Before washing away any uncombined proteins, the modified *β*-glucosidase and ferritin were incubated in the Ni- nitrilotriacetic acid (Ni-NTA) column for over 1 h to bind KG and HE. Unbound excess HE was eluted using buffers containing low concentrations of imidazole. High concentrations of imidazole were used to elute the protein complex from the Ni-NTA column. The KG-HE complex was then dialyzed in a pH 7.5 buffer to remove the imidazole.

### 2.7. Comparison of Enzymatic Properties between Free Enzyme and Immobilized Enzyme

The properties of the *β*-glucosidase activity were investigated with the chromogenic substrate *p*-nitrophenyl-α-D-glucopyranoside (*p*NPG) (Aladdin, Shanghai, China). Hydrolysis of *p*NPG presented at a concentration of 5 mM, was assayed by the release of *p*-nitrophenol (*p*NP) spectrophotometrically at a wavelength of 400 nm [25,26]. Changes in absorbance were converted to mmol *p*NP according to the calibration curve (Figure S1) prepared with known *p*NP concentrations. The enzyme (10 µL) was mixed with an equal volume of *p*NPG in 180 µL of 50 mM imidazole-potassium acid phthalate buffer (pH 6.0), and incubated for 20 min at 45 °C. Subsequently, termination of reaction was performed by adding 600 µL of 1 M Na_2_CO_3_ to the reaction system. A total of 200 µL of solution was detected at 400 nm to calculate the relative enzyme activity.

For enzyme kinetic determination, reactions were performed depending on the optimal conditions of free or immobilized *β*-glucosidase. Reactions were carried out using 0.1 mM to 1.0 mM *p*NPG as the substrate in a total volume of 0.8 mL. The released *p*-nitrophenol was monitored continuously at 400 nm by a microplate reader (BioTek, Winooski, VT, USA).

### 2.8. Reusability of Immobilized Enzyme

To evaluate the effects of an applied magnetic field, the KG-HE complex solutions were placed within a magnetic field at 4 °C. All analyses were carried out in triplicate. The enzyme activity of the KG-HE complex prior to the introduction of the magnetic field was used as the baseline value. Magnetic immobilized enzymes were pulled to the magnetic field over 24 h and the supernatant, which should contain any nonmagnetic immobilized enzymes, was analyzed for residual enzymatic activity. After three usage cycles, each followed by exposure to a magnetic field, relative enzyme activities in the supernatant and magnetic immobilized enzymes were obtained, and the retention rates of relative enzyme activities were calculated.

## 3. Results and Discussion

### 3.1. Preparation and Purification of HE and KG

SDS-PAGE was applied to analyze each step of protein expression of HE and KG (Figure S2). The supernatant of HE and KG was purified by ammonium sulfate precipitation and affinity chromatography using Ni-NTA, respectively. For HE, there was an obvious target band at 24 kDa in soluble fraction after expression. Moreover, SDS-PAGE showed that the fraction at 40% saturation of ammonium sulfate precipitation was the optimal condition (Figure S2B), and the fraction of 40% was used for further purification. As it contains histidine-tag that can bind to the Ni-NTA resin, KG can be easily purified by affinity chromatography. A protein band corresponding to 55 kDa verified the successful purification of KG (Figure S2C).

**Figure 2:**
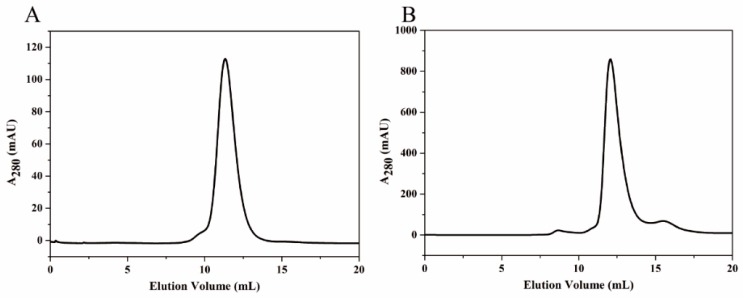
Size exclusion chromatography (SEC) of HE (**A**) and KG (**B**). Elution was monitored at 280 nm.

Protein HE or KG was further purified by SEC equipped with a Superdex^TM^ 200 10/300 GL column. As detected at 280 nm, pure HE eluted at V = 9.5 mL, whereas the KG eluted at V = 12.0 mL (Figure 2). The purified proteins were stored in 4 °C for later use.

### 3.2. Characterization of Magnetoferritin

**Figure 3:**
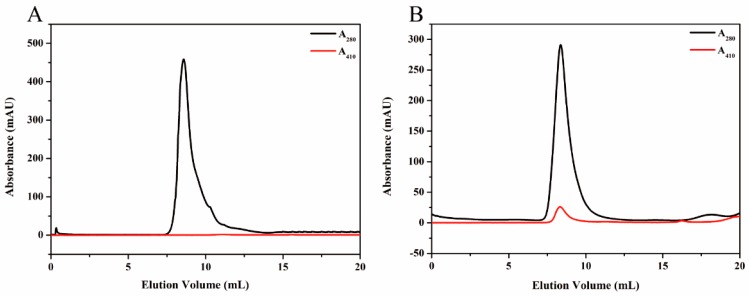
SEC of HE (**A**) before mineralization reaction and (**B**) after mineralization. The black lines represent the protein absorption at 280 nm and the red lines represent the iron oxide mineral absorption at 410 nm.

In the process of magnetoferritin preparation, the synthesis was carried out within an anaerobic chamber to prevent possible oxidation. During the synthesis of magnetoferritin, the color of the reaction solution gradually changed from colorless (empty apoferritin) to brown (filled ferritin). After centrifugation, the clear supernatant was collected. The synthesized magnetoferritin was purified by SEC and sucrose density gradient (SDG) ultracentrifugation. SDG was used to separate magnetoferritin from any empty apoferritin. In details, magnetoferritin was characterized and purified by SEC with detected wavelength at 280 nm (protein) and 410 nm (iron oxide mineral). There was an obvious absorption at 410 nm that appeared in the chromatogram of magnetoferritin, suggesting that iron oxide mineral was synthesized successfully in the cavity of the HE (Figure 3). Furthermore, SDG showed that ferritins with and without mineralization were stayed at approximately 41% and 20% of sucrose concentration, respectively (Figure S3), which demonstrated that ferritins were separated successfully using the SDG ultracentrifugation method.

**Figure 4:**
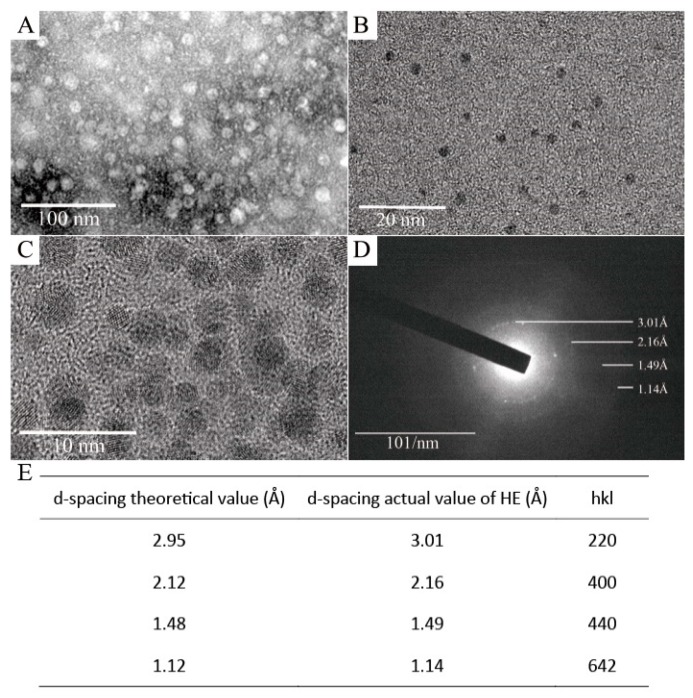
(**A**) Transmission electron microscopy (TEM) image of magnetoferritin of HE stained with 1% uranyl acetate. (**B**) TEM image of magnetoferritin of HE without staining. (**C**) High-resolution transmission electron microscopy (HRTEM) image of magnetoferritin of HE. (**D**) Selected area electron diffraction (SAED) image of magnetoferritin of HE. (**E**) Theoretical and actual d-spacings for magnetoferritin of HE.

To investigate the structure of magnetoferritin, transmission electron microscopy (TEM), high resolution transmission electronic microscopy (HRTEM), selected area electron diffraction (SAED), and Raman spectroscopy were applied. For TEM analyses, a small volume of magnetoferritin solution was treated with and without 1% uranyl acetate for TEM observations (JEM-1200EX). The results showed that HE exhibited an identical shell-like structure, with an exterior diameter of ~12 nm, after staining (Figure 4A). In the absence of uranyl acetate, apparent iron cores appeared in the inner cavities of HE with a diameter of ~4 nm, indicating the presence of Fe_3_O_4_ nanoparticles formed in the protein cavity (Figure 4B). Thus, HE could not only self-assemble into a shell structure, but could also be loaded with magnetic Fe_3_O_4_ nanoparticles.

HRTEM was used for further characterization. Biological materials are sensitive to an impinging electron beam. Therefore, all observations and image acquisitions were performed using exposures of <1 s to prevent damage to the ferritin molecules from the electron beam. Figure 4C shows typical lattice striations suggesting that Fe_3_O_4_ particles were successfully encapsulated within the inner cavity of HE ferritin. In addition, SAED of magnetic HE yielded *d* spacings of 2.95, 2.12, 1.48, and 1.12 Å (Figure 4D), corresponding to the (220), (400), (440), and (642) family of lattice planes, respectively, for γ-Fe_2_O_3_ or Fe_3_O_4_.

To further characterize the mineralization of ferritin, Raman experiments was carried out. Figure S4 showed the Raman trace of magnetoferritin. The target Raman shifts of magnetoferritins for γ-Fe_2_O_3_ (365 cm^−1^(T_2g_), 511 cm^−1^ (E_g_), and 700 cm^−1^ (A_1g_)) and Fe_3_O_4_ might be encapsulated within the cavity of the nanoparticles due to the overlapping peaks.

### 3.3. Formation of KG-HE Protein Complex

**Figure 5:**
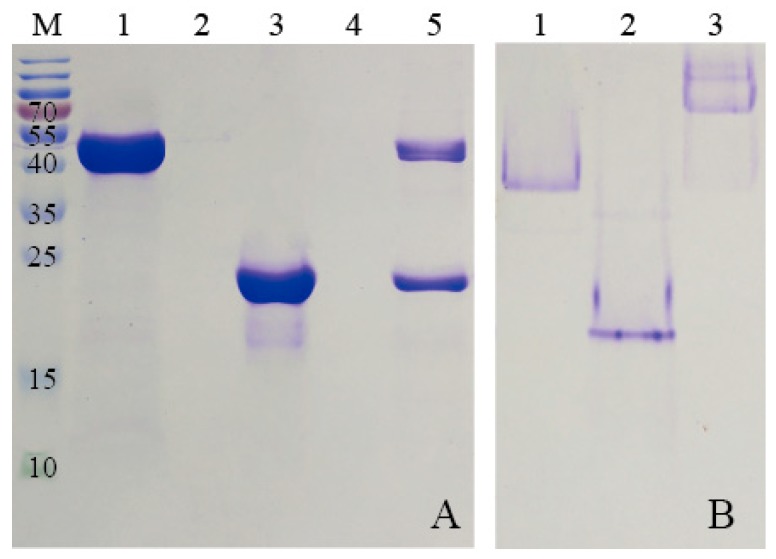
(**A**) Sodium dodecyl sulfate-polyacrylamide gel electrophoresis (SDS-PAGE) showing the interactions between HE and KG. Lane M: protein marker. Lane 1, KG; lane 2, elution fraction; lane 3, HE; lane 4, elution fraction; and lane 5, elution of KG-HE. (**B**) Non-denaturing polyacrylamide gel electrophoresis (Native PAGE) showing the interactions between HE and KG. Lane 1, HE; lane 2, KG; lane 3, KG-HE complex.

The N-terminal His-tag sequence of KG was used to immobilize the KG-HE protein complex on a Ni-NTA column for assembly and purification. In detail, the N-terminal His-tag of KG could bind to the Ni-NTA, and other proteins lacking His-tag would be removed in the buffer washing step. HE (lacking His-tag) was added into the Ni-NTA column which contained KG, and the E-coil of the ferritin could be captured by K-coil of the enzyme KG. Moreover, another washing step was used to remove free HE and finally the purified KG-HE complex was obtained. Characterization of the KG-HE protein complex was carried out by sodium dodecyl sulfate-polyacrylamide gel electrophoresis (SDS-PAGE) and non-denaturing polyacrylamide gel electrophoresis (Native PAGE). The results are shown in Figure 5. SDS-PAGE showed that KG-HE protein complex was composed of two subunits with molecular weights (MWs) of 55 and 24 kDa, respectively (Figure 5A), suggesting that the combination of ferritin and enzyme was successful. Native PAGE showed that the MW of the KG-HE complex was greater than that of either of the two proteins after binding (Figure 5B). These results indicate that the E-coil peptide that fused to the C-terminus of human H chain ferritin are presumably exposed on the surface of ferritin nanoparticles with a flop conformation, [23] and that the enzyme is positioned on the surface of ferritin due to interactions between the K- and E-coils.

### 3.4. Enzymatic Properties Analysis

**Figure 6:**
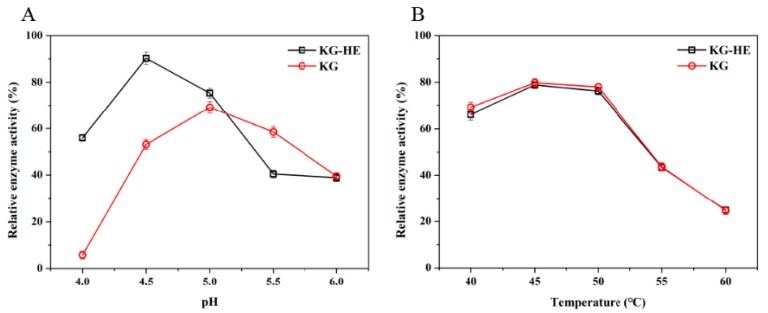
Effect of pH (**A**) and temperature (**B**) on free *β*-glucosidase (KG) and immobilized *β*-glucosidase (KG-HE). Black lines present relative enzyme activity of the KG-HE and red lines present relative enzyme activity of the KG. Data were expressed as mean ± standard deviation (n = 3).

The effect of pH and temperature on the activity of KG (free enzyme) and KG-HE (immobilized enzyme) were also evaluated. The KG showed greater activity at pH 5.0 than that of at other pHs (Figure 6A). However, the KG-HE showed a slightly different property as compared with the free one in which the enzyme activity of the KG-HE was observed to peak at pH 4.5 (Figure 6A). Moreover, it could be indicated that the enzyme of KG and KG-HE displayed the highest enzymatic activities at 45 °C (Figure 6B). The optimal temperature of the enzyme remained the same after immobilization, while the optimal pH changed only slightly, from 5.0 to 4.5. Under these optimal conditions, the specific activities of the enzyme were 78.1 U/mg and 79.0 U/mg for free and immobilized enzyme, respectively, demonstrating that immobilization did not result in a loss of enzyme activity. In addition, enzyme kinetics were determined using *p*-nitrophenyl-α-D-glucopyranoside (*p*NPG) as a substrate. The *K_m_* and *k_cat_* values were calculated by fitting the data to the Michaelis–Menten equation using SigmaPlot 12.5. The kinetic parameters *K_m_* and *k_cat_* values for KG and KG-HE were 1.26 mM and 65.26 s^−1^ and 1.44 mM and 70.55 s^−1^, respectively. To compare the catalytic effectiveness of enzymes, the *k_cat_* and *K_m_* values for KG and KG-HE were calculated to be 51.79 s^−1^·mM^−1^ and 48.99 s^−1^·mM^−1^, respectively. The results showed that immobilization on ferritin had almost no effect on the enzyme kinetics (Table 2).

### 3.5. Reusability of Immobilized Enzyme

**Figure 7:**
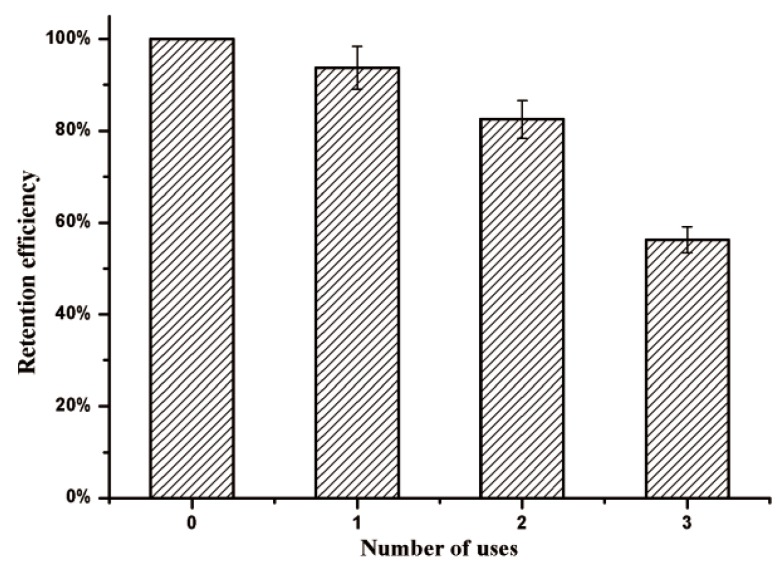
Retention efficiency of magnetic immobilized enzyme after three usage cycles.

Immobilization can facilitate enzyme recycling in catalytic process. The immobilized *β*-glucosidase was recycled by magnetic separation and reused three times. Figure 7 showed the changes in the enzymatic activities of KG-HE with increasing usage at the condition of pH 4.5, 45 °C, for 20 min. The immobilized enzyme maintained approximately 93.8% activity after the first use (Figure 7). KG-HE exhibited continuous loss in enzymatic activity with the repeat use, with a retention rate of 82.5% and 56.3%, respectively. Some reports have suggested that the modification of the enzyme structure, product inhibition, protein denaturation, or inactivation of enzyme would result ingradual loss of enzyme activity after a few cycles [27]. According to the results of enzymatic properties analyses comparing the free and immobilized enzyme, we found that immobilizing enzyme onto the surface of magnetoferritin did not influence the catalytic property of enzyme. Thus, the loss in retention efficiency was caused by protein denaturation of ferritin during the process of magnetic adsorption and catalytic reaction. In addition, the affinity between the two fractions that originated from the coiled coils was relatively weak and the immobilized enzyme may be lost which caused the decrease in the retention efficiency. In this study, the separation and recycling of the immobilized enzyme were efficient mainly due to the effect of magnetic nanoparticles.

## 4. Conclusions

In summary, ferritin can be functionalized with the addition of an E-coil and subsequently used to immobilize *β*-glucosidase which contains a K-coil. The two-stranded α-helical coiled-coil structure was the important motif to link two proteins together. As functionalized, paramagnetic particles with bound enzyme could be easily gathered for recycling under a magnetic field, providing a means of loading a variety of molecules onto the outer surface of ferritin. Magnetoferritin was successfully synthesized and applied to immobilize enzyme, achieving the goal of the enzyme reuse. After three times usage, it was observed that the retention rate was 93.8%, 82.5%, and 56.3%, respectively. In addition, the immobilized KG exhibited similar enzymatic properties and kinetic parameters as free KG. The strategy of enzyme immobilization developed in this work can be applied in general to many other objectives.

## Figures and Tables

**Table 1:** Amino acid sequences of E- and K-coil.

Coiled Coil	Amino Acid Sequence
E-coil	EIAALEKEIAALEKEIAALEK
K-coil	KIAALKEKIAALKEKIAALKE

**Table 2:** Catalytic activities of KG and KG-HE.

Enzyme	*k_cat_* (s^−1^)	*K_m_* (mM)	*k_cat_*/*K_m_* (s^−1^·mM^−1^)
KG	65.26	1.26	51.79
KG-HE	70.55	1.44	48.99

## Supplementary Materials

The following are available online at https://www.mdpi.com/2079-4991/9/11/1558/s1, Table S1: DNA primers used for the construction of the HE and KG protein, Figure S1: Standard curve of *p*-nitrophenyl, Figure S2: SDS-PAGE analyses, Figure S3: (A) Magnetoferritin purified by SDG. (B) UV absorbance of the sample after sucrose density gradient ultracentrifugation separation, Figure S4: Raman spectroscopy of magnetoferritin of HE.
