# Supplementary material for: Peptide-Mediated Immobilization on Magnetoferritin for Enzyme Recycling
Source: Nanomaterials (Basel). 2019 Nov 2;9(11):1558. doi: 10.3390/nano9111558 (PMC6915604; doi:10.3390/nano9111558)
Supplement: Supplementary file 1 [file nanomaterials-09-01558-s001.pdf]

nanomaterials

Supplementary Material

Peptide-Mediated Immobilization on Magnetoferritin for Enzyme Recycling

Yu Zhang [1,2,*], Yixin Dong [1,2], Jinhua Zhou [1,2], Ying'ao Hu [1,2], Xun Li [1,2] and Fei Wang [1,2]

[1] Jiangsu Provincial Key Lab for the Chemistry and Utilization of Ago-Forest Biomass, College of Chemical Engineering, Nanjing Forestry University, Nanjing 210037, China; kaydong417@163.com (Y.D.); 13770559414@163.com (J.Z.); hya8147213@163.com (Y.H.); xunlee@njfu.edu.cn (X.L.); hgwf@njfu.edu.cn (F.W.)

[2] Jiangsu Co-Innovation Center of Efficient Processing and Utilization of Forest Resources, Nanjing Forestry University, Nanjing 210037, China

* Correspondence: yuzhang@njfu.edu.cn; Tel.: +86-25-85427635; Fax: +86-25-85418873

Table S1. DNA primers used for the construction of the HE and KG protein.

**HE forward 5′-3′**	**Group 1:**<br>GAAAAAGAAATCGCGGCCCTAGAGAAGGAAATCGCGGCTCT<br>GGAAAAACTCGAGCACCACCACCAC<br>**Group 2:**<br>CTCGAGCACCACCACCAC
**HE reverse 5′-3′**	**Group 1:**<br>TAAGGCCGCGATCTCAGAACCACCGCCACCACTACCGCCGCC<br>CCCGCTTTCGTTATCAGAGTCACCC<br>**Group 2:**<br>TTTTTCCAGAGCCGCG
**KG forward 5′-3′**	GGCGGTGGAGGCTCCGGGGGTGGCGGTTCAGGTGGTGGTGGC<br>TCCATGGAAAAACTGCGCTTTCC
**KG reverse 5′-3′**	TTCTTTCAGTGCCGCAATTTTCTCTTTAAGCGCCGCAATTTTTTC<br>CTTGAGTGCTGCGATTTTGTGGTGGTGGTGATGATGCA

Note: Primers of Group 1 were used to construct ferritin with C-terminal E-coil sequence in vector pET-20b. Primers of Group 2 were used to remove the His-tag in vector pET-20b.

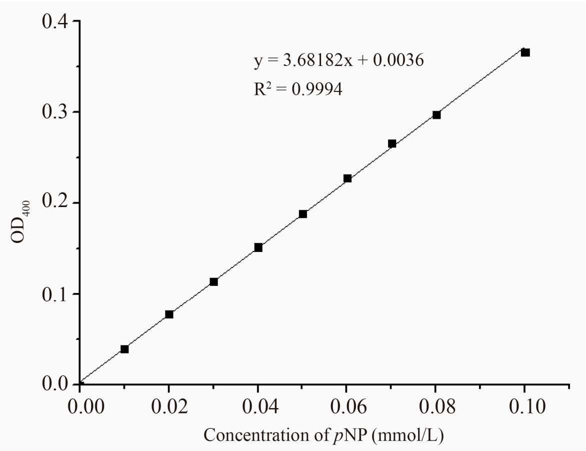

Figure S1. Standard curve of *p*-nitrophenyl.

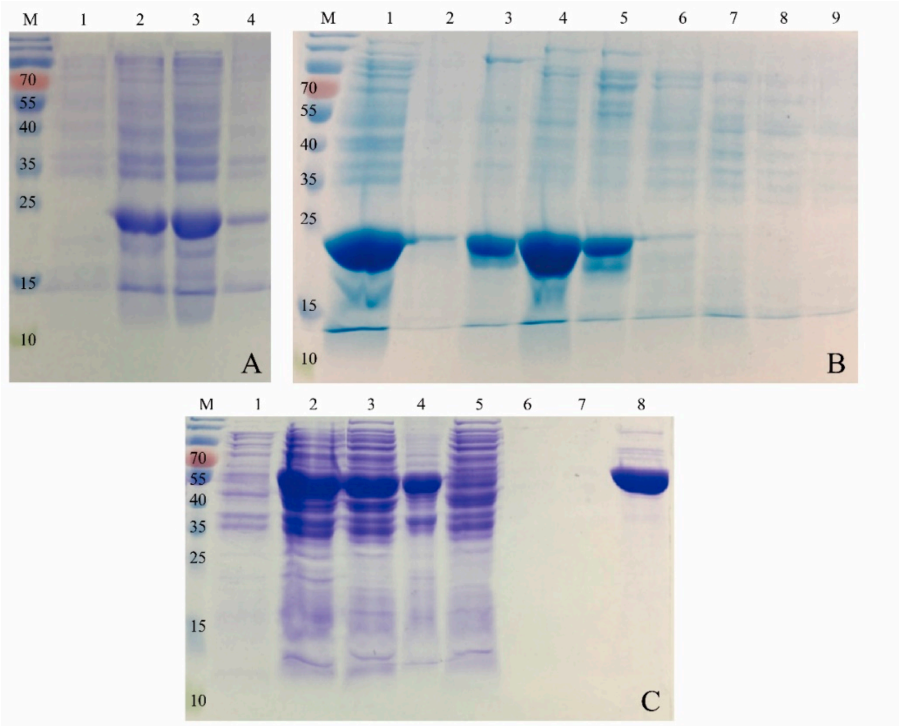

Figure S2. SDS-PAGE analyses. (A). SDS-PAGE of HE. Lane M: protein marker. Lane 1: before induction. Lane 2: after induction. Lane 3: soluble fraction. 4: insoluble fraction; (B). SDS-PAGE of ammonium sulfate precipitation of HE. Lane M: protein marker. Lane 1: soluble fraction. Lane 2: 20% ammonium sulfate. Lane 3: 30% ammonium sulfate.4: 40% ammonium sulfate. Lane 5: 50% ammonium sulfate. Lane 6: 60% ammonium sulfate. Lane 7: 70% ammonium sulfate. Lane 8: 80% ammonium sulfate. Lane 9: 90% ammonium sulfate; (C). SDS-PAGE of KG. Lane M: protein marker. Lane 1: before induction. Lane 2: after induction. Lane 3: soluble fraction. Lane 4: insoluble fraction. Lane 5: supernatant after Ni-NTA binding. Lane 6: flow-through (lysis buffer). Lane 7: flow-through (wash buffer). Lane 8: Elution.

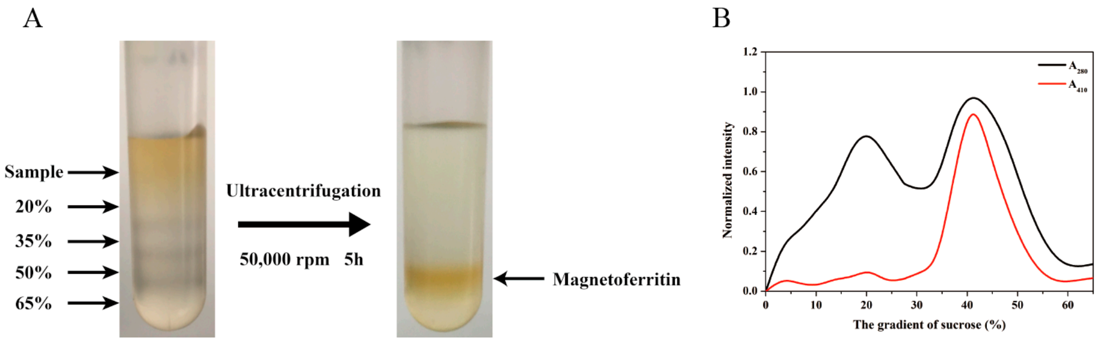

Figure S3. (A) Magnetoferritin purified by SDG. (B) UV absorbance of the sample after sucrose density gradient ultracentrifugation separation.

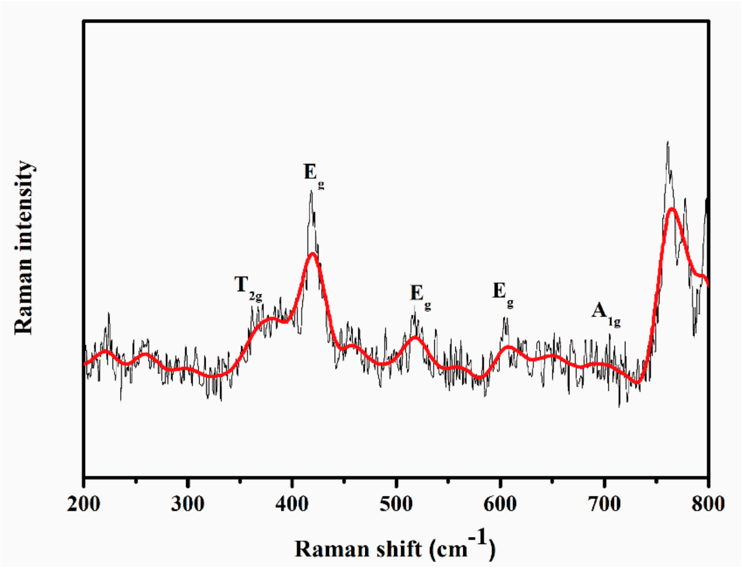

Figure S4. Raman spectroscopy of magnetoferritin of HE. There were Raman shifts of 365 (T_{2g}), 414 (E_g), 511 (E_g), 615 (E_g), 700 (A_{1g}). The Raman shifts of α-Fe_2O_3 are 229 (A_{1g}), 249 (E_g), 295 (E_g), 302 (E_g), 414 (E_g), 500 (A_{1g}), 615 (E_g), 660 (LOE_u), the Raman shifts of γ-Fe_2O_3 are 365 (T_{2g}), 511 (E_g), 700 (A_{1g}), and the Raman shifts of Fe_3O_4 are 310 (T_{2g}), 554 (T_{2g}), 672 (A_{1g}).
